# Therapeutic Hypothermia Improves Hind Limb Motor Outcome and Attenuates Oxidative Stress and Neuronal Damage in the Lumbar Spinal Cord Following Cardiac Arrest

**DOI:** 10.3390/antiox9010038

**Published:** 2020-01-01

**Authors:** Ji Hyeon Ahn, Tae-Kyeong Lee, Bora Kim, Jae-Chul Lee, Hyun-Jin Tae, Jeong Hwi Cho, Yoonsoo Park, Myoung Cheol Shin, Taek Geun Ohk, Chan Woo Park, Jun Hwi Cho, Seongkweon Hong, Joon Ha Park, Soo Young Choi, Moo-Ho Won

**Affiliations:** 1 Department of Biomedical Science, Research Institute for Bioscience and Biotechnology, Hallym University, Chuncheon 24252, Korea; jh-ahn@hallym.ac.kr; 2 Department of Neurobiology, School of Medicine, Kangwon National University, Chuncheon 24341, Korea; xorud312@naver.com (T.-K.L.); nbrkim17@gmail.com (B.K.); anajclee@kangwon.ac.kr (J.-C.L.); 3 Bio-Safety Research Institute, College of Veterinary Medicine, Chonbuk National University, Iksan 54596, Korea; anatotae@gmail.com (H.-J.T.); uribugi@naver.com (J.H.C.); 4 Department of Emergency Medicine, School of Medicine, Kangwon National University, Chuncheon 24341, Korea; pyoonsoo@naver.com (Y.P.); dr10126@naver.com (M.C.S.); otgotg11@gmail.com (T.G.O.); bonaeboa@naver.com (C.W.P.); cjhemd@kangwon.ac.kr (J.H.C.); 5 Department of Surgery, School of Medicine, Kangwon National University, Chuncheon 24341, Korea; skhong1@kangwon.ac.kr; 6 Department of Anatomy, College of Korean Medicine, Dongguk University, Gyeongju 38066, Korea; jh-park@dongguk.ac.kr

**Keywords:** antioxidants, asphyxial cardiac arrest, hypothermia, lumbar spinal cord, motor neurons, neuroprotection, oxidative stress

## Abstract

Hypothermia enhances outcomes of patients after resuscitation after cardiac arrest (CA). However, the underlying mechanism is not fully understood. In this study, we investigated effects of hypothermic therapy on neuronal damage/death, microglial activation, and changes of endogenous antioxidants in the anterior horn in the lumbar spinal cord in a rat model of asphyxial CA (ACA). A total of 77 adult male Sprague–Dawley rats were randomized into five groups: normal, sham ACA plus (+) normothermia, ACA + normothermia, sham ACA + hypothermia, and ACA + hypothermia. ACA was induced for 5 min by injecting vecuronium bromide. Therapeutic hypothermia was applied after return of spontaneous circulation (ROSC) via rapid cooling with isopropyl alcohol wipes, which was maintained at 33 ± 0.5 °C for 4 h. Normothermia groups were maintained at 37 ± 0.2 °C for 4 h. Neuronal protection, microgliosis, oxidative stress, and changes of endogenous antioxidants were evaluated at 12 h, 1 day, and 2 days after ROSC following ACA. ACA resulted in neuronal damage from 12 h after ROSC and evoked obvious degeneration/loss of spinal neurons in the ventral horn at 1 day after ACA, showing motor deficit of the hind limb. In addition, ACA resulted in a gradual increase in microgliosis with time after ACA. Therapeutic hypothermia significantly reduced neuronal loss and attenuated hind limb dysfunction, showing that hypothermia significantly attenuated microgliosis. Furthermore, hypothermia significantly suppressed ACA-induced increases of superoxide anion production and 8-hydroxyguanine expression, and significantly increased superoxide dismutase 1 (SOD1), SOD2, catalase, and glutathione peroxidase. Taken together, hypothermic therapy was found to have a substantial impact on changes in ACA-induced microglia activation, oxidative stress factors, and antioxidant enzymes in the ventral horn of the lumbar spinal cord, which closely correlate with neuronal protection and neurological performance after ACA.

## 1. Introduction

Vascular spinal disorders, non-traumatic spinal cord injuries, originate from tumors or cardiovascular disorders, and the spinal cord is vulnerable to ischemic damage [1]. Global ischemia after cardiac arrest (CA) causes a temporary interruption of the blood supply to the spinal arteries and results in neuronal damage in the spinal cord, which can lead to severe clinical symptoms, such as paralysis and sensory dysfunction [2]. Studies on damage in the spinal cord after asphyxial CA (ACA), which is apparently different from spinal cord ischemia, are still lacking compared to studies on spinal cord injury after spinal cord ischemia. Until now, inconsistent results in the distribution pattern of neuronal damage in the lumbar spinal cord after CA have been reported. One study shows damage of neurons in the dorsal horn of the gray matter and damage in the white matter [3], and another study shows damage of all neurons in the gray matter [4]. In addition, studies on damage in the spinal cord after ACA are still lacking compared to studies on spinal cord injury after spinal cord ischemia.

It is well known that both primary insult and secondary injury reactions after spinal cord injury result in tissue damage and cause neurological dysfunction [5]. Ischemia-reperfusion injury in the central nervous system triggers a complex series of pathophysiological events, including ATP depletion, reactive oxygen species (ROS) generation, intracellular calcium overload, and activation of inflammatory cascades that lead to neuronal death/loss [6]. 

It has been proven that hypothermic therapy can effectively protect multiple injuries of the brain and spinal cord [7]. In a swine model of CA, previous studies have shown that mild hypothermia has protective effects against ischemic damage in the brain and demonstrated that reduction of oxidative stress, enhancement of antioxidant enzyme expressions [8], inhibition of brain edema, proinflammatory cytokine [9], and complement activation [10] are underlying mechanisms for cerebral neuroprotection after applying hypothermia. 

Although many previous studies have reported on the positive effects of hypothermia as a neuroprotective approach after CA, in the context of spinal cord ischemia after CA, little is known about the possible neuroprotective mechanisms of therapeutic hypothermia. Recently, we reported that motor neurons in the lumbar spinal cord of the rat were dead or lost at 1 day after ACA, but therapeutic hypothermia protected the neurons at 1 day after ACA [11]. Based on these findings, the aim of this study was to identify pathological changes in the lumbar spinal neurons over time after spinal cord injury following ACA, and, in addition, to investigate related mechanisms of hypothermic therapy to develop mechanism-based neuroprotective strategies for CA-induced ischemic spinal cord injury.

## 2. Materials and Methods

### 2.1. Experimental Animals and Groups 

We purchased 56 male Sprague–Dawley rats (16 weeks of age; body weight 320–350 g) from Central Lab Animal Inc. (Seoul, Republic of Korea). They had been maintained in pathogen-free conditions under temperature of 23 °C and humidity of 60%. The experimental protocol of this study was approved by the Institutional Animal Care and Use Committee at Kangwon University (approval no. KW-180124-1). For animal handling and care, we abided by the Guide for the Care and Use of Laboratory Animals (The National Academies Press, 8th Ed., 2011). 

Rats were divided into five groups as follows: (1) the normal group (*n* = 5), which was given nothing; (2) the sham group subjected to an ACA operation and treated with normothermia (sham/normothermia group, *n* = 15), which was given identical anesthetic and surgical procedures without induction of ACA/cardiopulmonary resuscitation (CPR) operation. Body temperature was controlled at 37 ± 0.5 °C for 4 h after return of spontaneous circulation (ROSC). Five rats were sacrificed at 12 h, 1 day, and 2days, respectively; (3) the ACA/normothermia group (*n* = 21), which was given an ACA/CPR operation. Body temperature was controlled at 37 ± 0.5 °C for 4 h after ROSC. Seven rats were sacrificed at 12 h, 1 day, and 2 days, respectively, after ROSC; (4) the sham/hypothermia group (*n* = 15), which was given identical anesthetic and surgical procedures without induction of an ACA/CPR operation. Body temperature was controlled at 33 ± 0.5 °C for 4 h after ROSC. Five rats were sacrificed at 12 h, 1 day, and 2 days, respectively; and (5) the ACA/hypothermia group (*n* = 21), which was given an ACA/CPR operation. Body temperature was controlled at 33.0 ± 0.5 °C for 4 h after ROSC. Seven rats were sacrificed at 12 h, 1 day, and 2 days, respectively, after ROSC. 

### 2.2. Induction of ACA and CPR 

ACA induction was performed according to published protocols [12,13,14]. In short, rats in each group were anesthetized with a mixture of 2.5% isoflurane in 33% oxygen and 67% nitrous oxide. The rats were endotracheally intubated with a 14-gauge cannula and mechanically ventilated to preserve respiration through a rodent ventilator (Harvard Apparatus, Holliston, MA, USA). Peripheral oxygen saturation (SpO_2_) was monitored by an oxygen saturation probe of pulse oximetry (Nonin Medical Inc., Plymouth, MN, USA) attached on the left foot. Mean arterial pressure (MAP) was monitored by cannulation with a PE-50 catheter to the left femoral artery. The electrocardiogram (ECG) was monitored by electrocardiographic probes (GE healthcare, Milwaukee, WI, USA) placed in the limbs. Body (rectal) temperature was maintained at 37 ± 0.5 °C with a heat blanket. These data were continuously monitored. At 5 min after stabilization, vecuronium bromide (2 mg/kg) (Reyon Pharmaceutical, Seoul, Republic of Korea) was intravenously administered by right femoral vein cannulation. The anesthesia and mechanical ventilation was discontinued, and the endotracheal tube was disconnected from the ventilator. ACA was confirmed when MAP was below 25 mmHg, and a subsequent pulseless electric activity (PEA) was observed [15,16]. Usually, ACA was confirmed at about 3–4 min after the discontinuation of ventilation following vecuronium bromide injection in this study. There were no significant differences in characteristics, including body weight, anesthesia duration for preparation, and values of MAP, among the groups (data not shown).

After 5 min of ACA, CPR was initiated according to published protocols [12,13,14]. In short, the ventilator was reconnected with 100% oxygen, and epinephrine (0.005 mg/kg) (Dai Han Pharm, Seoul, Republic of Korea) and sodium bicarbonate (1 meq/kg, Daewon Pham, Seoul, Republic of Korea) was intravenously administered. Manual chest compression was done at a rate of 300/min for ROSC until MAP reached 60 mmHg, and ECG activity was observed [17,18]. (If ROSC was not detected, half of the injected amount of epinephrine was administered with 1 min of CPR. However, the rats that underwent CPR for a third time were excluded from this experiment.) 

### 2.3. Hypothermic Therapy

After ROSC, hypothermic therapy was provided by surface cooling with isopropyl alcohol wipes, ice packs, an electrical fan, and a cooling blanket according to published protocols [11,19]. Body temperature in the hypothermia group was maintained at 33 ± 0.5 °C for 4 h after ROSC and monitored by a rectal temperature sensor. Body temperature in the sham/normothermia and ACA/normothermia groups were maintained at 37 ± 0.5 °C by using a heating pad and lamps. Once each rat was hemodynamically stable and spontaneously breathed (usually 1 h after ROSC), the catheter was removed, and the animal extubated. The rats of the sham/hypothermia and ACA/hypothermia groups were re-warmed to 37 ± 0.5 °C for 30 min by using a warming blanket and hot pad; thereafter, all animals were kept in a thermal incubator (Mirae Medical Industry, Seoul, Republic of Korea) to maintain body temperature (37.0 ± 0.5 °C). 

### 2.4. Evaluation of Motor Function

Neurological function for motor function of the hind limbs was evaluated based on the Tarlov Scale [20] with modification on 1 day after ACA. Motor function of the hind limbs was graded as follows: 0, complete paraplegia with no movement of the hind limbs; 1, paraplegia and slight movement of the hind limbs (poor function, weak antigravity movement only); 2, good movement of the hind limbs but unable to stand without support (some function with good antigravity strength, but inability to draw legs under body or hope); 3, able to stand but unable to walk normally (ability to draw legs under body and hope, but not normally); and 4, complete recovery (normal movement).

### 2.5. Tissue Preparation for Histological Analyses

Tissue preparation was done at sham, 12 h, 1 day, and 2 days after ACA according to our published method [11]. All animals were anesthetized with sodium pentobarbital (60 mg/kg, i.p., JW Pharmaceutical, Seoul, Republic of Korea) and perfused transcardially with 0.1 M phosphate-buffered saline (PBS, pH 7.4) followed by 4% paraformaldehyde (in 0.1 M PB) (pH 7.4). Lumbar spinal cords were removed and post-fixed in the same fixative for 6 h. The lumbar spinal cord tissues were cryoprotected by infiltration with 25% sucrose for 8 h. Thereafter, the cord tissues were frozen and serially sectioned into 25 µm coronal sections in a cryostat (Leica, Wetzlar, Germany).

### 2.6. Cresyl Violet (CV) and Fluoro-Jade B (FJB) Histofluorescence Staining

To elucidate hypothermic effect on neuronal damage or death after ACA, CV staining and F-J B histofluorescence staining were carried out according to previously described methods [21]. Briefly, for CV (a marker for Nissl’s body) staining, the sections were stained with solution of 1.0% (w/v) CV acetate (Sigma-Aldrich, St. Louis, MO, USA) for 15 min at room temperature, and the stained sections were washed and dehydrated in serial ethanol baths. 

FJB (a high affinity fluorescent marker of neurodegeneration) histofluorescence staining was used to detect neuronal degeneration. The sections were immersed in solution of 0.0004% F-J B (Histochem, Jefferson, AR, USA). The immersed sections were washed and placed on a slide warmer (50 ± 1 °C) to be reacted and dehydrated. 

### 2.7. Immunohistochemistry 

Immunohistochemistry was performed to investigate (1) neuroinflammation by using ionized calcium binding adapter molecule 1 (Iba-1), (2) oxidative stress by using 8-hydroxy-2′ -deoxyguanosine (8-OHdG, a marker of oxidative stress), and (3) endogenous antioxidants by using SOD1, SOD2, catalase (CAT), and glutathione peroxidase (GPX). The sections were immunohistochemically stained according to our published protocol [22]. Briefly, the tissue sections were treated with 0.3% hydrogen peroxide (H_2_O_2_) in PBS for 30 min and then in 10% normal donkey serum in PBS for 30 min. The treated sections were incubated with each diluted primary antibody overnight at 4 °C as follows: rabbit anti-Iba1 (a marker for microglia) (1:800, Wako, Japan), goat anti-8-OHdG (1:200, Millipore, Billerica, MA, USA), rabbit anti-SOD1 (1:1000, Millipore, Billerica, MA, USA), rabbit anti-SOD2 (1:1000, Abcam, Cambridge, MA, USA), rabbit anti-CAT (1:500, Abfrontier, Seoul, Korea), and rabbit anti-GPX (1:500, Calbiochem, USA) were used as primary antibodies. Thereafter, the reacted sections were exposed to biotinylated goat anti-rabbit or rabbit anti-goat IgG (1:200, Vector, Burlingame, CA, USA) and streptavidin peroxidase complex (1:200, Vector, Burlingame, CA, USA). Finally, the reacted sections were visualized with a solution of 3,3’-diaminobenzidine tetrahydrochloride in 0.1 M Tris–HCl buffer (pH 7.2).

To establish the specificity of each immunostaining, each negative control test was done with pre-immune serum instead of each primary antibody. The test showed no immunoreactivity in the sections (data not shown).

### 2.8. Dihydroethidium (DHE) Fluorescence Staining 

To examine in situ production of superoxide anion, the oxidative fluorescent dye DHE (Sigma-Aldrich, St. Louis, MO, USA) was used to detect superoxide anion radical according to a published protocol [23]. Briefly, the sections were equilibrated in Krebs-HEPES buffer (130 mM NaCl, 5.6 mM KCl, 2 mM CaCl_2_, 0.24 mM MgCl_2_, 8.3 mM HEPES, 11 mM glucose, pH 7.4) for 30 min at 37 °C. The equilibrated sections were applied with a fresh buffer containing DHE (10 μmol/L) and incubated in a light-protected humidified chamber for 2 h at 37 °C.

### 2.9. Data Analyses

To quantitatively analyze neuronal death/loss or protection, five sections of the anterior horn of the lumbar spinal cord were selected with 120 μm intervals in each animal. CV- and FJB-positive neurons were counted as previously described [24]. Briefly, digital images of the cells were obtained using a BX53 upright microscope (Olympus, Japan) equipped with digital camera (DP7) (Olympus, Japan) connected to PC monitor. The cells were counted in a 500 μm × 500 μm square at the anterior horn of the lumbar spinal cord. The cell counts were evaluated by averaging total numbers by using an image analyzing system (software: Optimas 6.5) (CyberMetrics, Scottsdale, AZ, USA).

The relative fluorescence intensity of DHE was measured according to our published method [23]. In brief, digital images were captured by using a BX53 upright microscope (Olympus, Japan) with an excitation wavelength of 520–540 nm. The DHE fluorescence intensity was analyzed by using Image-pro Plus 6.0 software (Media Cybernetics, MD, USA) and calibrated as %, with the sham group designated as 100%. 

To quantitatively analyze the immunoreactivity of the Iba-1, 8-OHdG, SOD1, SOD2, CAT, and GPX immunoreactive structure, each digital image was taken as per the above-mentioned method and analyzed according to our published method [25]. Briefly, immunoreactivity of each image was evaluated on the basis of an optical density (OD). OD was obtained after the transformation of the mean gray level of each immunoreactive structure by using the formula OD = log (256/mean gray level). The background was taken from the areas adjacent to the measured areas. Finally, each OD was compared as a ratio of a relative optical density (ROD). ROD was calibrated as %, with the sham group designated as 100% by using Adobe Photoshop (version 8.0) (San Jose, CA, USA) and analyzed by using NIH Image software (version 1.59) (NIH, Bethesda, MD, USA).

### 2.10. Statistical Analysis 

All statistical analyses were performed with SPSS software (version 15.0, SPSS Inc., Chicago, IL, USA) and expressed as mean ± SEM. The significance of differences between the groups was assessed using one-way analysis of variance followed by a post-hoc Tukey test. Differences were considered significant at *p* < 0.05.

## 3. Results

### 3.1. Survival Rate by Hypothermia

**Figure 1:**
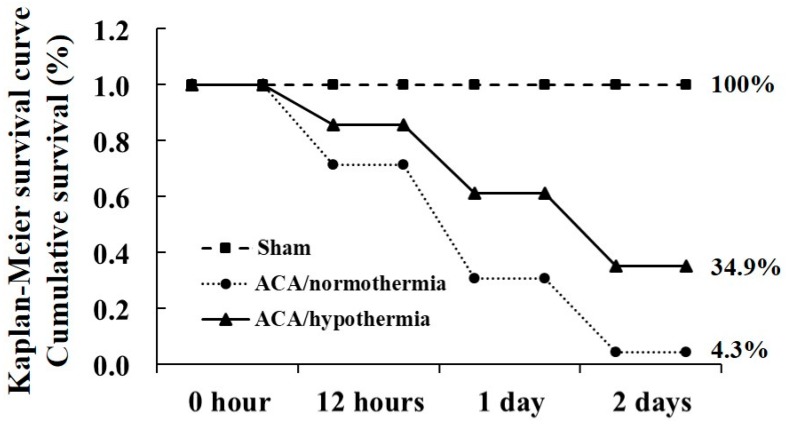
Cumulative survival rate (log-rank test, *p* < 0.05) in the sham, ACA/normothermia, and ACA/hypothermia groups using Kaplan–Meier analysis for 2 days after asphyxial cardiac arrest (ACA). The ACA/hypothermia group shows a higher survival rate than the ACA/normothermia group (*n* = 5 and 7 in sham and ACA group, respectively).

Survival rate of the experimental rats was confirmed by Kaplan–Meier analysis for 2 days after ACA (Figure 1). In the normal and sham/normothermia and sham/hypothermia groups, survival rate was 100%. In the ACA/normothermia group, survival rate was 71.4%, 30.6%, and 4.3% at 12 h, 1 day, and 2 days, respectively, after ACA. However, in the ACA/hypothermia group, survival rate was increased compared to that in the ACA/normothermia group: 85.7%, 61.2%, and 34.9% at 12 h, 1 day, and 2 days, respectively, after ACA.

### 3.2. Motor Deficit Score by Hypothermia

**Figure 2:**
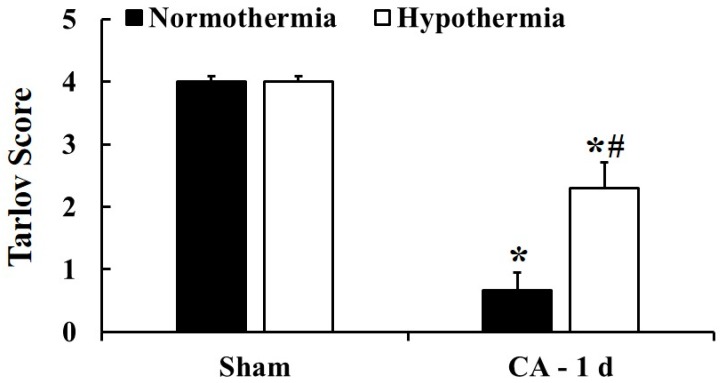
Neurological evaluation for hind limb motor function in the sham/normothermia, sham/hypothermia, ACA/normothermia, and ACA/hypothermia groups at 1 day after ACA. The ACA/hypothermia group shows a milder neurological deficit than the ACA/normothermia group. A high score represents good motor function. The bars indicate the means ± SEM (*n* = 5, 7, and 7 for the sham, ACA/normothermia, and ACA/hypothermia group, respectively; * *p* < 0.05 vs. each sham group; ^#^ *p* < 0.05 vs. the ACA/normothermia group).

Neurological evaluation was conducted to examine motor function of the hind limbs according to the modified Tarlov score at 1 day after ACA (Figure 2). In the sham/normothermia and sham/hypothermia groups, the rats showed completely normal neurological function (4 and 4 points, respectively) at 1 day after ACA. In the ACA/normothermia group, the rats showed normal neurological function before ACA, but showed a clear dysfunction (0.67 point) in the hind limbs at 1 day after ACA. However, the rats in the ACA/hypothermia group significantly showed a mild neurological deficit (2.3 point) compared to those in the ACA/normothermia group. 

### 3.3. Neuroprotection by Hypothermia

The data from the normal group was not shown because the results were not different from the sham/normothermia group. In addition, the sham/normothermia and sham/hypothermia groups showed similar results at 12 h, 1 day, and 2 days after ROSC; therefore, the data were shown at 12 h after the sham operation.

#### 3.3.1. CV- and FJB-Positive Cells 

**Figure 3:**
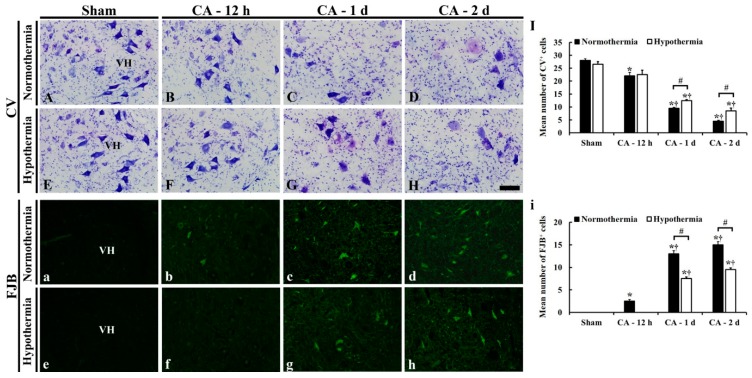
CV staining (**A**–**H**) and FJB fluorescence staining (**a**–**h**) in the lumbar ventral horn of sham/normothermia (**A**,**a**), ACA/normothermia (**B**–**D**,**b**–**d**), sham/hypothermia (**E**,**e**), and ACA/hypothermia (**F**–**H**,**f**–**h**) group. In the sham/normothermia and sham/hypothermia groups, many CV-positive and no FJB-positive motor neurons were found. In the ACA/normothermia group, CV-positive cells are gradually damaged and reduced in number from 12 h until 2 days after ACA. A few FJB-positive neurons are shown at 12 h after ACA; thereafter, many FJB-positive neurons are detected until 2 days after ACA. In the ACA/hypothermia group, CV-positive cells are much less damaged at 12 h, 1 day, and 2 days after ACA than those in the ACA/normothermia groups. FJB-positive cells are not shown at 12 h after ACA, and their number at 1 and 2 days was lower than that in the ACA/normothermia group. VH, ventral horn. Scale bar = 100 μm. (**I,i**) Mean number of (**I**) CV- and (**i**) FJB-positive motor neurons in the ventral horn. The bars indicate the means ± SEM (*n* = 5 and 7 for the sham and ACA groups; * *p* < 0.05 vs. the sham group; ^#^ *p* < 0.05 vs. the ACA/normothermia group; ^†^ *p* < 0.05 vs. the previous time-point group).

CV staining and FJB fluorescence staining was performed to examine cellular change and degeneration, respectively, in the ventral horn of the lumbar spinal cord in all the groups (Figure 3). In the sham/normothermia and sham/hypothermia groups, large and pyramid-like CV-positive motor neurons were easily found throughout in ventral horn of the lumbar spinal cord (Figure 3A,E). In these groups, FJB-positive cells were not detected in the ventral horn (Figure 3a,e).

In the ACA/normothermia groups, the distribution pattern of CV-positive cells in the ventral horn was altered from 12 h after ACA, showing that the CV-positive cells contained dark and polygonal nuclei (Figure 3B–D). In addition, the number of CV-positive cells in the ACA/normothermia groups was significantly decreased with time compared to that in the sham/normothermia group: The mean number of CV-positive cells in the ventral horn was 22 ± 1.4, 9.5 ± 0.4, and 4.5 ± 0.3, respectively, at 12 h, 1 day, and 2 days after ACA (Figure 3I). FJB-positive degenerated neurons were detected in the ventral horn of the sham/normothermia group at 12 h after ACA. Their number was gradually and significantly increased until 2 days after ACA (Figure 3b–d): The mean number of FJB-positive cells was 2.5 ± 0.3, 13.2 ± 0.7, and 15.6 ± 0.6, respectively, at 12 h, 1 day, and 2 days after ACA (Figure 3i).

In the ACA/hypothermia groups, the distribution pattern of CV-positive cells observed at 12 h after ACA was similar to that in the sham/hypothermia group (Figure 3F), and no FJB-positive cells were found (Figure 3f). At 1 and 2 days after ACA, the number of CV-positive cells were higher (Figure 3I), whereas the number of FJB-positive cells were lower (Figure 3i) than those in the ACA/normothermia groups, showing that many of the CV- and FJB-positive cells were much less damaged than the ACA/normothermia groups (Figure 3G,H,g,h).

#### 3.3.2. Reduced Microglia Activation by Hypothermia

**Figure 4:**
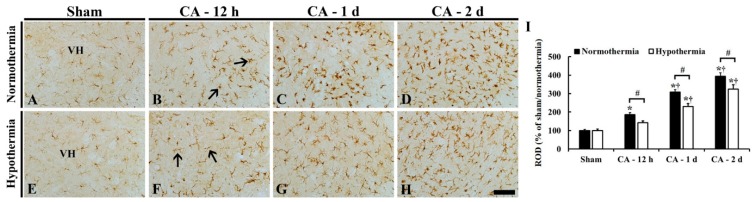
Iba-1 immunohistochemistry in the lumbar ventral horn of the sham/normothermia (**A**), ACA/normothermia (**B**–**D**), sham/hypothermia (**E**), and ACA/hypothermia (**F**–**H**) groups. In the ACA/normothermia group, Iba-1 immunoreactivity (arrows) is significantly increased from 12 h after ACA compared to that in the sham/normothermia group. However, in the ACA/hypothermia group, Iba-1 immunoreactivity (arrows) at 12 h after ACA is similar to that in the sham/hypothermia group; thereafter, Iba-1 immunoreactivity is significantly lower than that in the ACA/normothermia group. VH, ventral horn. Scale bar = 100 μm. (**I**) ROD as % of Iba-1 immunoreactive structures in the lumbar ventral horn. The bars indicate the means ± SEM (*n* = 5 and 7 for the sham and ACA groups; * *p* < 0.05 vs. each sham group; ^#^ *p* < 0.05 vs. the ACA/normothermia group; ^†^ *p* < 0.05 vs. the previous time-point group).

Iba-1 (a marker for microglia) immunohistochemistry was performed to examine microglia activation in the ventral horn of the lumbar spinal cord in the ACA/normothermia and ACA/hypothermia groups (Figure 4). In the sham/normothermia and sham/hypothermia groups, Iba-1 immunoreactive microglia were scattered in the lumbar ventral horn, and they had small cytoplasm and thin processes as a resting form (Figure 4A,E).

In the ACA/normothermia group, Iba-1 immunoreactive microglia had enlarged cytoplasm with thickened processes as an active form (Figure 4B–D). Iba-1 immunoreactivity in the microglia was significantly increased with time by about 90%, 210%, and 290%, respectively, at 12 h, 1 day, and 2 days after ACA compared to that in the sham/normothermia group (Figure 4I). 

In the ACA/hypothermia group, the activation of Iba-1 immunoreactive microglia was attenuated, showing that their Iba-1 immunoreactivity was significantly decreased about 50% at 12 h, 80% at 1 day, and 70% at 2 days after ROSC compared to that in the ACA/normothermia group (Figure 4F–H,I).

### 3.4. Reduced Oxidative Stress by Hypothermia

#### 3.4.1. DHE Fluorescence

**Figure 5:**
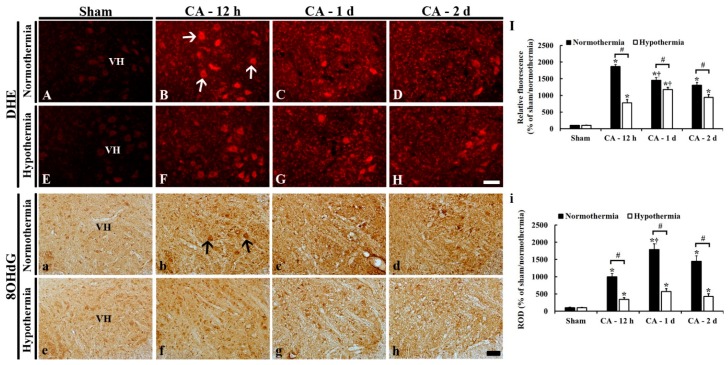
DHE fluorescence staining (**A**–**H**) and 8OHdG immunohistochemistry (**a**–**h**) in the lumbar ventral horn of the sham/normothermia (**A**), ACA/normothermia (**B**–**D**), sham/hypothermia (**E**), and ACA/hypothermia (**F**–**H**) groups. In the ACA/normothermia group, DHE fluorescence (arrows) and 8OHdG immunoreactivity (arrows) are significantly increased compared to that in the sham/normothermia, showing that each level is the highest at 12 h and 1 day, respectively, after ACA. However, in the ACA/hypothermia group, DHE fluorescence and 8OHdG immunoreactivity are significantly lower than that in the ACA/normothermia group. VH, ventral horn. Scale bar = 100 μm. (**I,i**) Relative DHE fluorescence (**I**) and ROD as % of 8OHdG immunoreactive structures (**i**) in the lumbar ventral horn. The bars indicate the means ± SEM (*n* = 5 and 7 for the sham and ACA groups; * *p* < 0.05 vs. each sham group; ^#^ *p* < 0.05 vs. the ACA/normothermia group; ^†^ *p* < 0.05 vs. the previous time-point group).

DHE fluorescence staining was performed to examine in situ superoxide anion level in the lumbar ventral horn in the ACA/normothermia and ACA/hypothermia groups (Figure 5A–I). In the sham/normothermia and sham/ hypothermia groups, like the finding in the normal group (data not shown), a weak superoxide anion signal was detected in the ventral horn (Figure 5A,E).

In the ACA/normothermia group, the superoxide anion level in the ventral horn was significantly increased by about 1760%, 1350%, and 1210%, respectively, at 12 h, 1 day, and 2 days after ACA, compared to that in the sham/normothermia group, showing that the level was the highest at 12 h after ACA (Figure 5B–D,I).

In the ACA/hypothermia group, the superoxide anion level in the ventral horn was significantly lower than that in the ACA/normothermia group; it was decreased about 1090% at 12 h, 280% at 1 day, and 370% at 2 days after ACA when compared to that in the ACA/normothermia group, showing that the level was the highest at 1 day after ACA (Figure 5F–H,I).

#### 3.4.2. 8OHdG Immunoreactivity

8OHdG immunohistochemistry was performed to examine oxidative nuclear stress in the lumbar ventral horn in the ACA/normothermia and ACA/hypothermia groups (Figure 5a–i). In the sham/normothermia and sham/hypothermia groups, 8OHdG immunoreactivity was shown in the nuclei of neuronal cells in the ventral horn, showing that 8OHdG immunoreactivity in both groups was similar (Figure 5a,e,i).

In the ACA/normothermia group, 8OHdG immunoreactivity was strong: 8OHdG immunoreactivity was significantly increased about 900% at 12 h, 1690% at 1 day, and 1350% at 2 days after ACA when compared to that in ACA/sham group (Figure 5b–d,i).

In the ACA/hypothermia group, 8OHdG immunoreactivity was significantly lower compared to that in the ACA/normothermia group (Figure 5f–h), showing that 8OHdG immunoreactivity at 12 h, 1 day, and 2 days after ACA was significantly decreased about 660%, 1220%, and 1020%, respectively, compared to that in the ACA/normothermia group (Figure 5i).

### 3.5. Increased Anti-Oxidant Enzymes by Hypothermia

**Figure 6:**
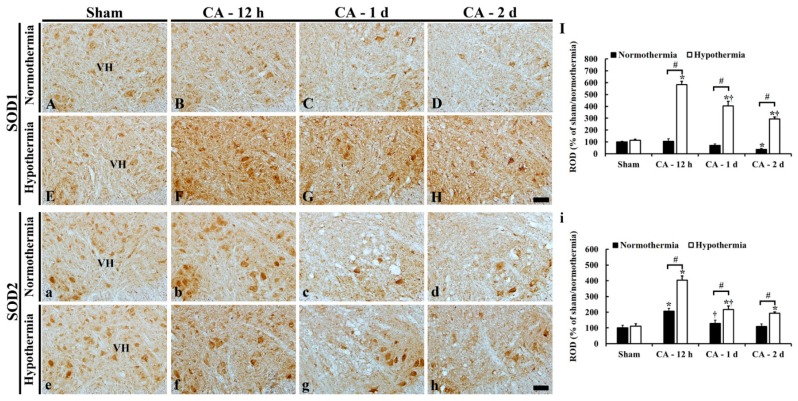
Immunohistochemistry of SOD1 (**A**–**H**) and SOD2 (**a**–**h**) in the lumbar ventral horn of the sham/normothermia (**A**,**a**), ACA/normothermia (**B**–**D**,**b**–**d**), sham/hypothermia (**E**,**e**), and ACA/hypothermia (**F**–**H**,**f**–**h**) groups. In the ACA/normothermia group, SOD1 immunoreactivity is slightly decreased with time after ACA. In the ACA/hypothermia group, SOD1 immunoreactivity is significantly increased compared to that in the ACA/normothermia group. SOD2 immunoreactivity in the ACA/normothermia group is increased at 12 h after ACA, thereafter SOD2 immunoreactivity is gradually decreased. In the ACA/hypothermia group, SOD2 immunoreactivity is significantly increased compared to that the ACA/normothermia group. VH, ventral horn. Scale bar = 100 μm. (**I**,**i**) ROD as % of SOD1 (**I**) and SOD2 (**i**) immunoreactive structures in the lumbar ventral horn. The bars indicate the means ± SEM (*n* = 5 and 7 for the sham and ACA group; * *p* < 0.05 vs. each sham group; ^#^ *p* < 0.05 vs. the ACA/normothermia group; ^†^ *p* < 0.05 vs. the previous time-point group).

**Figure 7:**
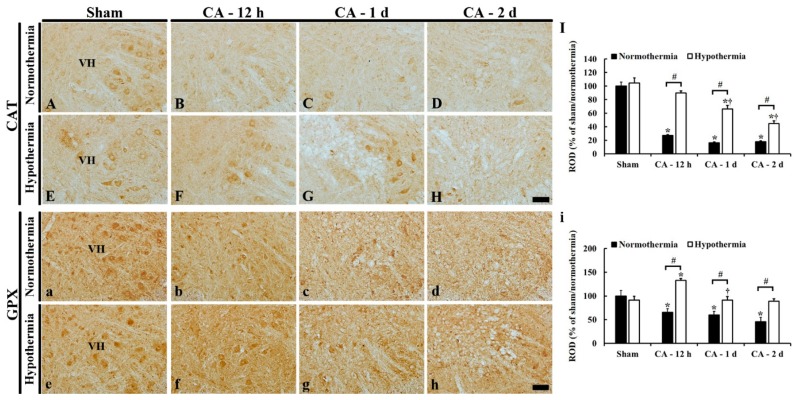
Immunohistochemistry of CAT (**A**–**H**) and GPX (**a**–**h**) in the lumbar ventral horn of the sham/normothermia (**A**,**a**), ACA/normothermia (**B**–**D**,**b**–**d**), sham/hypothermia (**E**,**e**), and ACA/hypothermia (**F**–**H**,**f**–**h**) groups. In the ACA/normothermia group, CAT immunoreactivity is dramatically decreased at 12 h after ACA and gradually decreased with time. In the ACA/hypothermia group, CAT immunoreactivity is similar to that in the sham/normothermia group, thereafter, CAT immunoreactivity is gradually decreased and very low at 2 days after ACA. GPX immunoreactivity in the ACA/normothermia group is gradually decreased with time. In the ACA/hypothermia group, GPX immunoreactivity at 12 h after ACA is significantly higher than that in the sham group, and GPX immunoreactivity is gradually reduced. VH, ventral horn. Scale bar = 100 μm. (**I**,**i**) ROD as % of CAT (**I**) and GPX (**i**) immunoreactive structures in the lumbar ventral horn. The bars indicate the means ± SEM (*n* = 5 and 7 for the sham and ACA group, * *p* < 0.05 vs. each sham group; ^#^ *p* < 0.05 vs. the ACA/normothermia group; ^†^ *p* < 0.05 vs. the previous time-point group).

SOD1, SOD2, CAT, and GPX immunohistochemistry was performed to examine changes in endogenous antioxidant enzyme expressions in the lumbar ventral horn of the ACA/normothermia and ACA/hypothermia groups (Figure 6 and Figure 7).

#### 3.5.1. SOD1 Immunoreactivity 

In all sham groups, SOD1 immunoreactivity was shown in neuronal somata in the lumbar ventral horn (Figure 6A,E).

In the ACA/normothermia group, SOD1 immunoreactivity in the ventral horn at 12 h after ACA was similar to that in the sham/normothermia group (Figure 6B,I). Thereafter, SOD1 immunoreactivity was gradually decreased, showing that SOD1 immunoreactivity at 1 day and 2 days after ACA was significantly decreased about 70% and 40%, respectively, compared to that in the sham/normothermia group (Figure 6C–I). 

In the ACA/hypothermia group, SOD1 immunoreactivity was significantly higher (about 480% at 12 h, 330% at 1 day, and 260% at 2 days) compared to that in the ACA/normothermia group (Figure 6F–I).

#### 3.5.2. SOD2 Immunoreactivity 

SOD2 immunoreactivity in all sham groups was also shown in neuronal somata in the lumbar ventral horn (Figure 6a,e).

In the ACA/normothermia group, SOD2 immunoreactivity at 12 h after ACA was significantly increased about 110% compared to that in the sham/normothermia group (Figure 6b,i). Thereafter, the increased SOD2 immunoreactivity was steadily reduced until 2 days after ACA (Figure 6c,d), showing that SOD2 immunoreactivity at 2 days after ACA was similar to that of the sham/normothermia group (Figure 6i). 

In the ACA/hypothermia group, SOD2 immunoreactivity at 12 h after ACA was significantly higher (about 200%) compared to that in the ACA/normothermia group (Figure 6f,i). Thereafter, the increased SOD2 immunoreactivity was gradually decreased until 2 days after ACA, showing that SOD2 immunoreactivity at 2 days after ACA was higher than that in the ACA/normothermia group (Figure 6g–i).

#### 3.5.3. CAT Immunoreactivity

In the sham/normothermia and sham/hypothermia groups, CAT immunoreactivity in the lumbar ventral horn was mainly found in the cytoplasm of neurons (Figure 7A,E).

In the ACA/normothermia group, CAT immunoreactivity was dramatically decreased at 12 h (about 30% of the sham/normothermia group); thereafter, more decreased (about 20% at 1 day and 20% at 2 days) compared to that in the sham/normothermia group (Figure 7B–D,I)

In the ACA/hypothermia group, CAT immunoreactivity at 12 h after ACA was significantly higher (about 60% of the ACA/normothermia group) compared to that in the ACA/normothermia group (Figure 7F,I); thereafter, CAT immunoreactivity was gradually decreased, showing that CAT immunoreactivity at 2 days after ACA was about 30% of the ACA/normothermia group (Figure 7G–I).

#### 3.5.4. GPX Immunoreactivity

In all sham groups, GPX immunoreactivity was also shown in the cytoplasm of neurons of the lumbar ventral horn (Figure 7a,e).

In the ACA/normothermia group, GPX immunoreactivity was significantly decreased at 12 h after ACA (about 70% of the sham/normothermia group) (Figure 7b,i); thereafter, GPX immunoreactivity was gradually decreased (about 60% at 1 day and 50% at 2 days after ACA compared to that of the sham/normothermia group) (Figure 7c,d,i).

In the ACA/hypothermia group, GPX immunoreactivity at 12 h after ACA was significantly higher (about 70%) compared to that in the ACA/normothermia group (Figure 7f,i) and gradually decreased (about 40% at 1 day and 40% at 2 days after ACA) compared to that in the ACA/normothermia group (Figure 7g–i).

## 4. Discussion

Induction of hypothermia after CA to protect brains has been extensively studied; however, few researches on the spinal cord protection and related mechanisms have been reported. So, in the present study, we investigated possible mechanisms of therapeutic hypothermia in the ACA-induced lumbar spinal cord ischemia by referring to previous studies. To the best of our knowledge, our current study is the first in evaluating mechanisms of efficacy of therapeutic hypothermia on neuroprotection in the ventral horn of the lumbar spinal cord.

Imaizumi et al. have reported that CA results in principal damage in the anterior horn of the spinal gray matter and permanent paraplegia [2]. In addition, ischemic susceptibility in the spinal cord increases caudally and is more prominent in the anterior horns than in the posterior horn [2]. Furthermore, it has been reported that ischemic vulnerability of the lumbar spinal region is considerably high compared to the other regions [4] since much energy may be required for extensive activity of motor neurons in the lumbosacral spinal region [26]. Lu et al. have reported that spinal cord ischemia by thoracic aortic occlusion induces histopathological changes in spinal neurons, such as eosinophilic or condensed cytoplasm, and a significant loss of neurons (NeuN-immunoreactive neurons) in the ventral horn of the lumbar spinal cord from 1 day (around 1–3 days) after spinal cord ischemia [27]. Consistent with the previously established ischemic damages of spinal motor neurons in the lumbar spinal cord after spinal cord ischemia, in our current study, severe and progressive neuronal degeneration was found in the ventral horn of the lumbar spinal cord at 1 day after ACA. 

In our current study regarding effects on the rats of the ACA/hypothermia group, neuronal loss was much milder and delayed in the ACA/hypothermia group than that in the ACA/normothermia group, although once the neurons began to damage, they progressed irreversibly. In addition, we found that therapeutic (systemic) hypothermia decreased mortality rate after ACA and ameliorated ACA-induced hind limb motor dysfunction, which is in agreement with our recent study [11]. In a rat model of ventricular fibrillation-induced CA, Ye et al. have reported that rapid and early systemic mild hypothermia (for 2 h) after resuscitation improves survival rate and neurological outcomes after ROSC [28], and Gong et al. have demonstrated that mild hypothermia (for 8 h) after ROSC improves microcirculatory blood supply and oxygen uptake, which is associated with cerebral protection [29]. On the other hand, in experimental animal models of spinal cord contusion, neuroprotective effects of hypothermia have been demonstrated by more preserved spinal neurons or tissues loss, much better locomotor ability, and higher potential amplitude in the somatosensory area [30,31,32]. These findings suggest that hypothermia has a significant neuroprotective effect on damaged spinal neurons, which is closely related to improvement of hind limb motor dysfunction (amelioration of hind limb motor dysfunction) due to damage/death of motor neurons in the ventral horn of the lumbar spinal cord, although the loss of spinal motor neurons may be propagated over time. 

After ROSC, a pathological condition considered is post cardiac arrest syndrome (PCAS), which involves brain injury, myocardial dysfunction, systemic ischemia/reperfusion response, and a persistent precipitating pathophysiological process [33,34]. Pathological changes between CA and ROSC may be related to the degree of neurological dysfunction. Microglia, resident immune cells in the central nervous system (CNS), are increased in number and morphologically activated at an early stage after spinal cord injury [35,36], which contributes to neuroinflammation and onset of neuronal damage by releasing large amount of inflammatory cytokines and ROS [37,38]. Similar to previous studies, our results showed that ACA resulted in significant microglia activation in the ventral horn of the lumbar spinal cord. Additionally, therapeutic hypothermia significantly ameliorated ACA-induced microglia activation in the lumbar ventral horn. This result is consistent with the previously established role of hypothermia in regulation of microglia-mediated inflammation in spinal cord injuries [39,40]. Therefore, these results support our current finding that therapeutic hypothermia resulted in attenuation of microglia activation in the ventral horn of the lumbar spinal cord after ACA.

It is well known that oxidative stress occurs due to rapid generation and accumulation of ROS and depletion of antioxidants, which is associated with secondary damage and inflammation in the CNS [41,42]. In the present study, we assumed that oxidative damage might be a primary factor to neuronal damage in the spinal cord after ACA and compared changes in oxidative stress (in situ superoxide anion level and 8OHdG) and endogenous antioxidants (SOD1, SOD2, CAT, and GPX) and microglia activation (Iba-1) in the lumbar ventral horn between the ACA/normothermia and ACA/hypothermia groups. As a result, we show that ACA-induced superoxide anion levels and 8OHdG immunoreactivity were significantly increased, and that antioxidant expressions were generally decreased. This finding is in line with previous investigations as follows: An immediate and significant increase in the superoxide anion level in the spinal gray matter after spinal cord contusion [43], and a preceding and progressive increase in 8OHdG expression in motor neurons before neuronal death after spinal cord ischemia [44] and in transgenic amyotrophic lateral sclerosis [45]. And these studies have suggested that superoxide anion and 8OHdG production are closely related to the death of ventral horn neurons in the spinal cord. Furthermore, it has been demonstrated that there is a significant reduction of endogenous SODs and CAT levels in the anterior horn of the spinal cord following spinal cord ischemia [43,46]. In the present study, application of 4 h systemic hypothermia significantly suppressed CA-induced increases of superoxide anion levels and 8OHdG immunoreactivity and significantly increased SOD1, SOD2, CAT, and GPX immunoreactivities in the lumbar ventral horn before ACA-induced neuronal death. Recently, antioxidant therapies containing enzymatic antioxidants (SOD, CAT, and GPX) have been developed for spinal cord injury because they can maintain the oxidation/reduction balance and diminish deleterious secondary injury cascades caused by excess of free radicals during onset of neuronal damage [47]. Additionally, several previous studies have demonstrated possible neuroprotective mechanisms of therapeutic hypothermia in spinal cord injury. Therapeutic hypothermia can decrease ROS generation and ameliorate inflammation in damaged spinal cords [5,48]. Taken together, therapeutic hypothermia can play important roles in decreasing the production of superoxide anions and nuclear oxidative stress, as well as in increasing endogenous antioxidants that lead to attenuation of ischemic damage of spinal motor neurons after ACA. 

In conclusion, our current results showed that ACA induced irreversible neuronal damage in the ventral horn in the lumbar spinal cord and that hypothermic therapy delayed or protected neuronal death from ACA-induced injury. This neuroprotective effect by hypothermic therapy may be closely related to amelioration of microglia activation, attenuation of oxidative stress, and enhancement of antioxidant enzyme expressions. Taken together, our results indicate that inhibiting oxidative stress and enhancing antioxidants enzymes can represent an effective strategy for treatment of spinal neuronal death/damage following ACA. Protection of spinal neurons from whole-body ischemia-reperfusion injury can improve motor function of lower extremities, which increases the quality of life of patients. Therefore, much more further studies must be done to investigate mechanisms of hypothermic therapy and to search for various drugs or materials that can cause hypothermia.
